# Time-resolved measurements of fast electron recirculation for relativistically intense femtosecond scale laser-plasma interactions

**DOI:** 10.1038/s41598-018-22422-6

**Published:** 2018-03-14

**Authors:** J. S. Green, N. Booth, R. J. Dance, R. J. Gray, D. A. MacLellan, A. Marshall, P. McKenna, C. D. Murphy, C. P. Ridgers, A. P. L. Robinson, D. Rusby, R. H. H. Scott, L. Wilson

**Affiliations:** 1 Central Laser Facility, STFC, Rutherford Appleton Laboratory, Chilton, Oxon, OX11 0QX UK; 2 York Plasma Institute, Department of Physics, University of York, York, YO10 5DD UK; 3 Department of Physics, SUPA, University of Strathclyde, Glasgow, G4 0NG UK

## Abstract

A key issue in realising the development of a number of applications of high-intensity lasers is the dynamics of the fast electrons produced and how to diagnose them. We report on measurements of fast electron transport in aluminium targets in the ultra-intense, short-pulse (<50 fs) regime using a high resolution temporally and spatially resolved optical probe. The measurements show a rapidly (≈0.5*c*) expanding region of Ohmic heating at the rear of the target, driven by lateral transport of the fast electron population inside the target. Simulations demonstrate that a broad angular distribution of fast electrons on the order of 60° is required, in conjunction with extensive recirculation of the electron population, in order to drive such lateral transport. These results provide fundamental new insight into fast electron dynamics driven by ultra-short laser pulses, which is an important regime for the development of laser-based radiation and particle sources.

## Introduction

The development of many cutting-edge applications in the field of ultra-high intensity laser-plasma interactions is highly dependent on progressing our understanding of fast electron generation and transport. The creation of high current beams of energetic electrons from ultra-high intensity laser-plasma interactions^1,2^ is the first stage in the process of creating hard X-ray sources^3,4^, high energy ions^5^ or warm dense matter (WDM) via isochoric heating^6^. Rapid isochoric heating of matter using fast electrons is also key to the fast ignitor scheme for inertial confinement fusion (ICF)^7^. Understanding exactly how relativistic electrons are transported away from the laser focal spot and into the bulk of a dense target remains a priority for the development of these science and application areas^8–10^.

A number of complementary techniques have been used to diagnose fast electron beam transport in dense targets in an attempt to characterise the electron beam temperature, flux and divergence. Of these key fast electron beam properties, both diagnosing and controlling the beam divergen ce is perhaps the greatest challenge, with previous experimental measurements^11^ indicating that the fast electron divergence half angle is on the order of 45° at intensities of ≈10^20^ Wcm^−2^. Current theory points to either the curvature of the critical surface at the front of the target or the influence of intense magnetic fields (possibly sourced from a Weibel-like instability) as the source of fast electron divergence^11,12^. However the growth of resistive magnetic fields inside the target bulk can subsequently alter the beam divergence as it propagates through the bulk plasma^13–15^. Almost all of the recent experimental work on this topic has been conducted on picosecond systems where the timescales are sufficient to allow magnetic fields and instabilities to develop significantly during the interaction. As the pulse length is reduced from ≈500 fs to <50 fs we might expect that the balance of these complex physical processes to be quite different. By performing experiments at this shorter pulse length range and with a temporally resolved diagnostic, an opportunity is presented to clarify the dominant processes for determining electron transport on sub-picosecond timescales.

**Figure 1:**
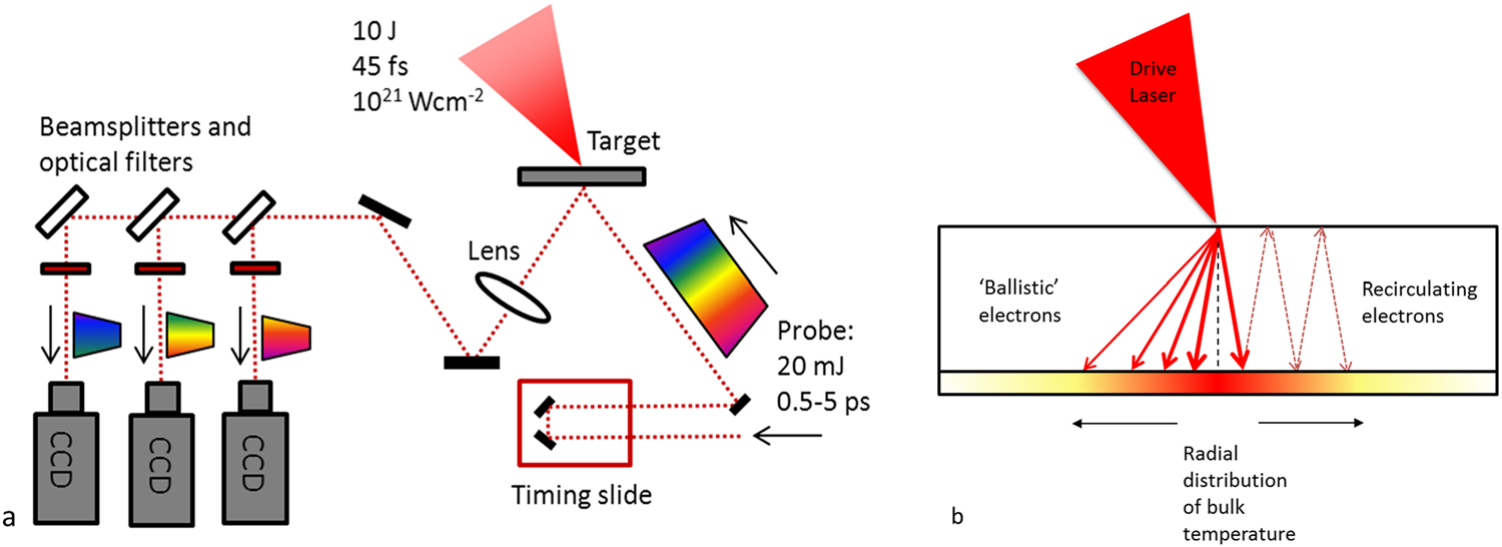
(**a**) Schematic of the rear surface optical probe layout for the experiment. (**b**) Schematic illustrating ballistic and recirculating model for electron transport in a solid target. Both models could result in a radial distribution of bulk temperature at the target rear surface, with the evolution of the heated region depending on the initial electron divergence angle and the degree of recirculation.

Diagnostic techniques can be broadly separated into those involving measurements of electrons inside the target and measurements of electrons (typically > 1 MeV) that have escaped the target. Direct measurements of electrons that have escaped the target give some indication of the electron distribution^16^ and divergence^17^ within it. However as these high energy electrons leave the rear surface they rapidly charge up the target, creating large electrostatic sheath fields that reflect all but the highest energy electrons inside the target. Since similar sheath fields are also produced at the target front side, fast electrons can recirculate^18^ inside the target (see Fig. 1b), driving lateral expansion away from the focal region^19^ and modifying the electron population that can escape^20^. Previous work has estimated that up to 99% of the fast electron population remains trapped in the target^21^ in this way. Electron recirculation has been attributed to significantly enhancing sheath-accelerated ions^22,23^ or x-ray yields^24^. Recirculation can also lead to enhanced heating of the bulk target material^25^, which in turn could affect subsequent electron transport owing to changes in the target resistivity^26^. However electron recirculation could also be an inhibiting factor in controlling fast electron divergence by disrupting collimating azimuthal magnetic fields inside the target^14^. The degrees to which electron recirculation and the initial electron injection profile affect subsequent transport physics clearly impacts many areas of laser-plasma sources and hence requires the development of diagnostic approaches more suited to spatially and temporally resolving electron transport inside the target.

X-ray spectroscopy^27^, Bremsstrahlung^28^ and Cu K-alpha techniques are often used to investigate aspects of fast electron beam divergence^29,30^ or refluxing dynamics^24,31^. While 2D spatially resolved (time integrated) K-alpha data can be obtained, careful analysis is required in order to relate measurements of K-alpha back to the original fast electron distribution, especially when buried layer targets are utilised where fields can build up at material interfaces. However, recent time-resolved measurements have provided spatially and temporally resolved data on a short-pulse interaction^32^.

Optical transition radiation (OTR) that is emitted as electrons cross the target-vacuum interface can be effective in characterising the fast electron divergence^33,34^ of the highest energy (>1 MeV) electrons. However, since OTR is emitted over a timescale similar to that of the laser drive pulse, temporally resolving the data is challenging.

Transverse (with respect to the target surface) optical probing has proven to be a relatively simple technique, that when matched with a suitable analytical or computational model can infer energy deposited at the rear surface of a target, which can then be used to determine the original fast electron parameters^35,36^. Temporal resolution is limited by the probe pulse duration and unless probing can be done in multiple directions, cylindrical symmetry has to be assumed in order to extract the data.

One promising alternative is the rear surface reflective optical probe. Instead of probing transversely across the target surface, measurements are made as a short-pulse of laser light reflects off the rear of the target surface, in the region opposite to the laser interaction on the front side. This technique has been used for long-pulse (ns), lower intensity experiments, to measure shock heating of plasmas for example^37,38^. Antici *et al*.^39^ applied a similar technique to high-intensity interactions (10^19^ Wcm^−2^), using a time-resolved optical probe to investigate bulk electron and fast electron populations. By using a chirped optical probe in conjunction with a spectrometer, they were able to characterise electron parameters at the rear surface with 4 ps temporal resolution. Chatterjee *et al*.^40^ have also used a complementary technique to measure fast electron-induced magnetic field evolution at the target rear surface.

Most applications of laser-accelerated radiation sources will require lasers that can operate at high repetition rates and within a small spatial footprint, requirements for which Ti:Sapphire systems are well-suited. As high repetition rate (~1 Hz), next-generation petawatt-class lasers come on-line, data on fundamental electron transport dynamics driven by short pulse systems (<50 fs) is crucial to underpinning application and scientific development. In this article we present time-resolved measurements of fast electron heating of Al foils in the ultra-high intensity (10^21^ Wcm^−2^), short pulse (40 fs) regime. This was achieved using a new rear surface probing technique^41^ that permits multiple time-resolved 2D measurements to be made for a single shot with temporal and spatial resolutions as low as 100 fs and 6 *μ*m respectively.

Based on our measurements and closely coupled simulations, we report that high energy electrons are accelerated into the target over a large half angle of ≈60°, with electron recirculation driving strong lateral transport of energy over areas significantly larger than the original laser focal spot and on timescales much longer than the pulse duration. These results will have significant impact on the development of fast electron guiding techniques; permitting greater beam control and higher fluxes for the optimisation of high repetition rate laser-based applications such as bright X-ray sources^42^ or radiation hardness testing^43^ of electronic equipment.

## Experimental Results

**Figure 2:**
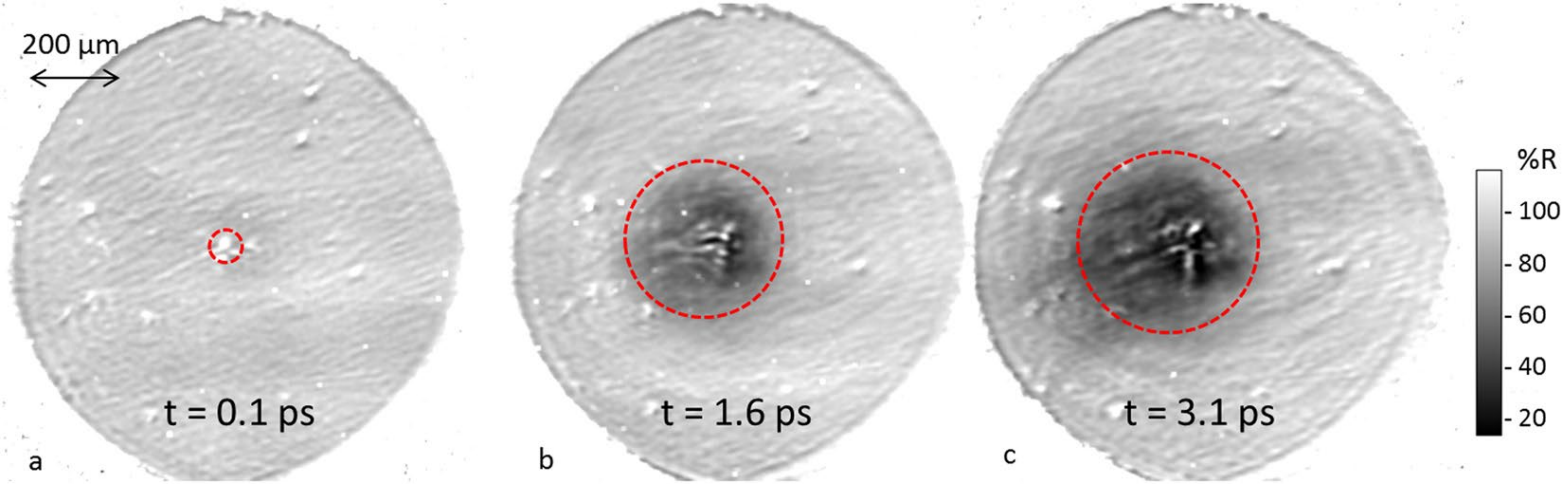
Experimental data showing reflectivity maps for a 50 *μ*m aluminium foil at 3 different probe times (same shot). For each time step a characteristic region of heating is illustrated with a dotted circle, corresponding to where the measured reflectivity drops below 90% of that of cold aluminium.

Figure 2 shows measurements of the spatial-intensity distribution of a laser pulse reflected from the rear surface of a 50 *μ*m Al foil, over three time steps, driven by a single pump beam focused to 10^21^ Wcm^−2^ at the target front surface (see Methods for more details). In order to enhance the quality of the data, the 50 *μ*m Al foil targets were polished prior to mounting in order to improve the surface quality by removing larger imperfections such as roll marks or scratches, although some imperfections remained. At the timing corresponding to the arrival of the main pulse (Fig. 2a), the reflectivity of the probe pulse can be seen to be broadly uniform across the target surface.

Figure 2b and c show the reflectivity of the rear surface of the foil 1.6 ps and 3.1 ps after the main interaction. A number of features are seen to evolve compared to the t = 0.1 ps image. A significant drop in target reflectivity is seen around the interaction point (close to the centre of the foil), extending out to 200 *μ*m in radius in Fig. 2c. OTR emission can also be seen centred on the interaction point for each time step, indicating where the highest energy electrons transition from the bulk target material into the vacuum.

**Figure 3:**
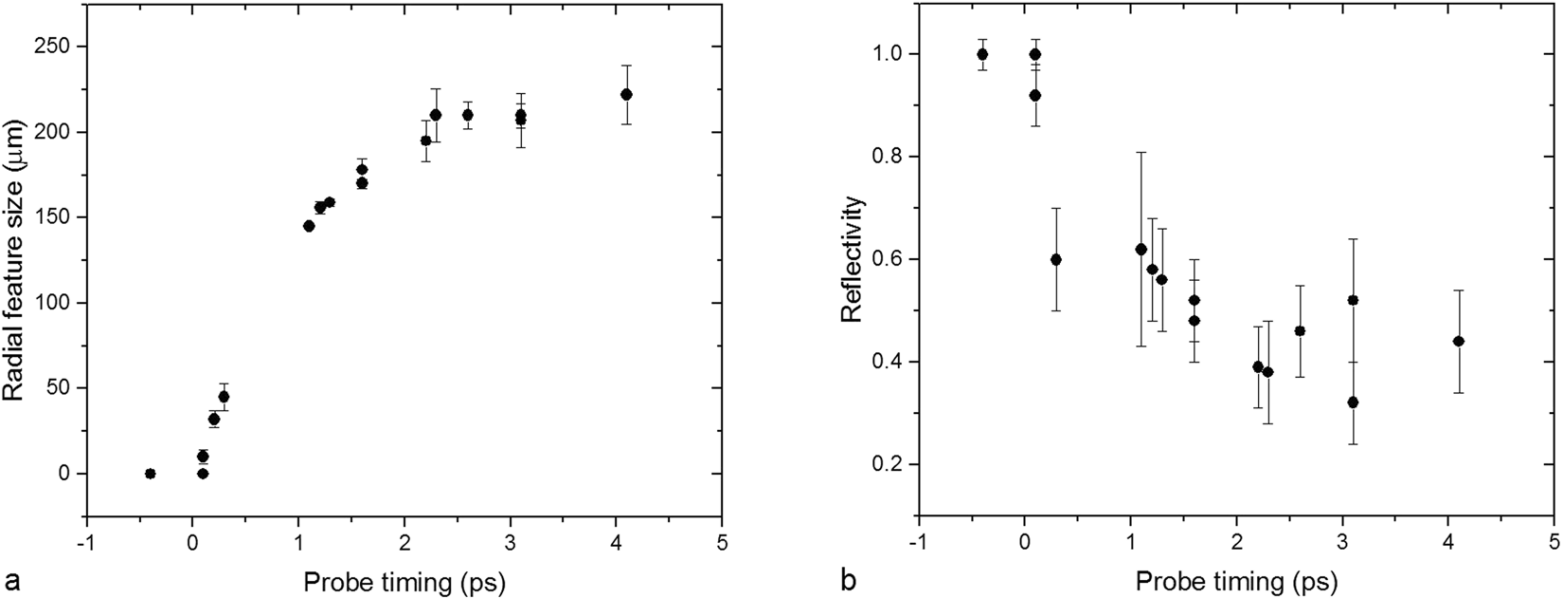
(**a**) Radial measurement of area of reduced reflectivity (<90%) as a function of probe time. (**b**) Minimum reflectivity recorded as a function of probe time. Error bars represent the variation in determining both the radial size and minimum reflectivity of the observed feature for each data point over multiple line outs.

The change in rear surface reflectivity for 50 *μ*m Al foils was measured over a range of time delays following the main interaction at the target front surface. For each shot, three time windows were obtained corresponding to three probing wavelengths (see Methods for details). For each probe image a vertical line out was taken through the central interaction region, from where the region of lower reflectivity was centred. For each data point a characteristic radial measurement (defined by the region where reflectivity falls below 90% that of the cold reflectivity) and minimum reflectivity value were extracted. The radial size of this dark feature is plotted against probe time in Fig. 3.

From Fig. 3a it can be seen that the spatial extent of the dark feature increases rapidly at early times (<1 ps), at a speed of ≈0.5 c before slowing at later times. The rapid nature of the initial expansion period is characteristic of a process driven by the generation of large currents of high energy electrons from the ultra-intense interaction of the pump pulse. The arrival of mega-ampere currents *I*_*f*_ at the target rear surface drives near-instantaneous Ohmic heating of the bulk material via the drawing of a low energy return current *I*_*b*_ ≈ *I*_*f*_^44^. Using the rigid beam model devised by Davies *et al*.^45^ the level of Ohmic heating would be expected to scale as:

1 $$\frac{dT}{dt}=\frac{\eta {J}_{f}^{2}}{C}$$

where *J*_*f*_ is the fast electron current density, *C* is the heat capacity (assumed constant) and *η* is the electrical resistivity scaling as:

2 $$\eta ={\eta }_{0}{(\frac{T}{{T}_{0}})}^{\alpha }$$

where *η*_0_ and *T*_0_ are the initial values and *α* < 1 for the low bulk temperatures (T < 10 eV) that will be considered here.

This rapid Ohmic heating increases the resistivity of the aluminium (for a Lee-More model^46^), resulting in a corresponding reduction of the reflectivity assuming a reflection of an electro-magnetic wave at a step-like density profile. It should be noted that at later times the assumption of a step-like density profile at the target rear surface may not apply, hence we would expect a proportion of the probe light to also be absorbed by non-linear processes.

In this model of fast electron-driven Ohmic heating the speed and change in reflectivity of the apparent radial expansion can be directly related to the electron transport inside the target foil. Assuming a ballistic injection of fast electrons from the laser focal region, an initial region of rear surface heating would be expected (as illustrated in Fig. 1), with the size of the heated region scaling primarily with the divergence angle of the electron beam. If we were to assume that the spatial profile of the observed heating is determined purely from the electron divergence angle then the data in Fig. 3 implies a divergence half-angle of >80°. Perez *et al*. highlighted that the fast electron divergence could appear almost isotropic in some situations, partly owing to how certain diagnostic data is interpreted^47^, however such measurements are the exception. In the time window presented in the data (4 ps), any electrons trapped within the target would have time to recirculate many times between the front and rear surfaces. This would have the effect of extending the region of heating radially as the recirculating electrons deposit additional energy in the bulk material. Hence care is required when interpreting such data, as demonstrated by Ridgers *et al*.^48^.

Some previous publications have presented evidence of lateral energy transport, commonly attributed to fast electron transport, although under different conditions^19,20,49^. A number of papers have reported on radial energy transport on femtosecond timescales at the target front surface during high intensity laser-plasma interactions^50–52^ where strong fields associated with the laser focal spot dominate transport. Metskes *et al*.^53^ have applied an optical probing reflectivity technique to map plasma formation and expansion on the target rear surface during high intensity (4 × 10^19^ Wcm^−2^) femtosecond scale interactions. Metskes *et al*. were able to observe a pre-pulse disrupted rear surface feature in addition to a darker electron-heated region at later times. We note that the measurements in the present paper exhibit no drop in reflectivity prior to the arrival of the main pulse, indicating little disruption from any laser pre-pulse.

Martonolli *et al*.^54^ made reflectivity measurements of the rear surface of aluminium targets with a picosecond class laser (t = 350 fs). Martonolli *et al*. attributed an expanding heated region to rapid energy deposition by the fast electron population within a few picoseconds followed by an unspecified transport process that occurs on timescales of tens of picoseconds, although the authors were limited by the low temporal resolution of the probe, preventing more detailed analysis of the initial heating phase. The high temporal resolution of the optical probe data presented here permits a new and important insight into fast electron transport and target heating at interaction intensities directly relevant for many laser-based applications.

## Simulations

In order to investigate the underpinning physics of the observed electron transport patterns at the target rear surface the 3D hybrid-PIC code Zephyros^55^ was used to simulate the effect of injecting a relativistic beam of fast electrons into a 50 *μ*m Al foil. The simulations were carried out using a box of 800 × 800 × 50 *μ*m (where 50 *μ*m was the target thickness) with a grid resolution set to 1 *μ*m. The cold aluminium target was initiated with a bulk temperature of 0.1 eV, and a resistivity curve determined by Davies *et al*.^56^ based on a Lee-More model^46^. The fast electron input parameters were defined to match those of the main Gemini laser pulse (10^21^ Wcm^−2^, 40 fs, 3 *μ*m FWHM focal spot). The electron temperature was set using the scaling of Haines *et al*.^57^, with the electron beam injected with an average injection half angle of 60 degrees, as derived from the scaling law of Green *et al*.^11^. In order to clarify the role of electron recirculation, two simulations were performed, with recirculation enabled and disabled.

As a first step in the analysis, the bulk electron temperature and resistivity of the rear surface of the aluminium target was extracted at various time steps during the simulation. Next the reflectivity of the bulk aluminium was calculated using Fresnel equations together with the incidence angle (*θ*_*i*_ = 30°) and polarisation (p) of the probe pulse:

3 $$ \% R={|\frac{{n}_{1}\sqrt{1-{(\frac{{n}_{1}}{{n}_{2}}\sin {\theta }_{i})}^{2}}-{n}_{2}\cos {\theta }_{i}}{{n}_{1}\sqrt{1-{(\frac{{n}_{1}}{{n}_{2}}\sin {\theta }_{i})}^{2}}+{n}_{2}\cos {\theta }_{i}}|}^{2}$$

where *n*_1_ and *n*_2_ are the refractive indices for a vaccum and the target material respectively and *n*_2_ is calculated from the aluminium resistivity *η* by:

4 $${n}_{2}=\sqrt{1+\frac{i}{{\omega }\eta {\varepsilon }_{0}}}$$

This reflectivity calculation can be considered valid for step-like density profiles, but at some point after the initial interaction, the expansion of the plasma into vacuum would become significant enough that absorption of the probe light in the plasma could not be neglected. In order to model the reflectivity of the probe more accurately the level of absorption of the probe light into a steep (but non-zero) plasma scale length was also considered. To provide a simple model for a steep scale length at the rear surface, the bulk temperature maps from Zephyros were used to calculate a time-dependant plasma expansion velocity (sound speed) and hence a time-dependant scalelength *L*_*n*_ where ${L}_{n}={n}_{e}\frac{dx}{d{n}_{e}}$.

At the typical intensity of the probe laser, and ultra-short scalelengths (≪1 *μ*m) predicted to be present during the experiment on <5 ps timescales, resonance absorption would be expected to be an additional absorption mechanism. Both Fresnel and resonance absorption^58^ were then combined to relate the observed drop in reflectivity of aluminium to the fast electron-sourced heating at the back of the target. Absorption by Inverse Bremsstrahlung was also included in the calculations, but found to be negligible at such short scalelengths.

**Figure 4:**
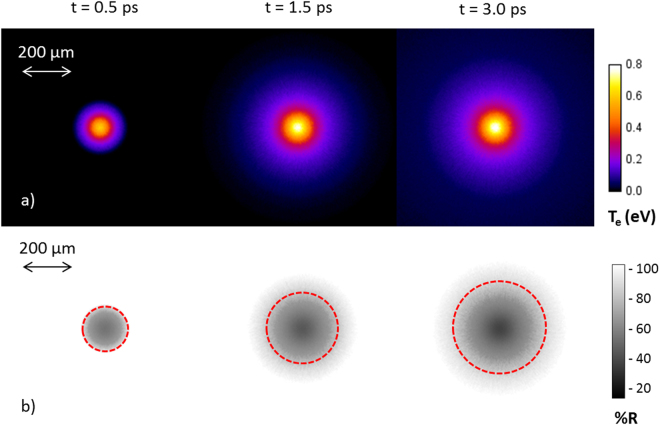
Simulated rear surface bulk temperatures (**a**) and calculated reflectivity (**b**) for an electron population injected with a 60 degree half angle with electron recirculation enabled. For the reflectivities a dotted circle marks the area within which the reflectivity drops below 90%.

**Figure 5:**
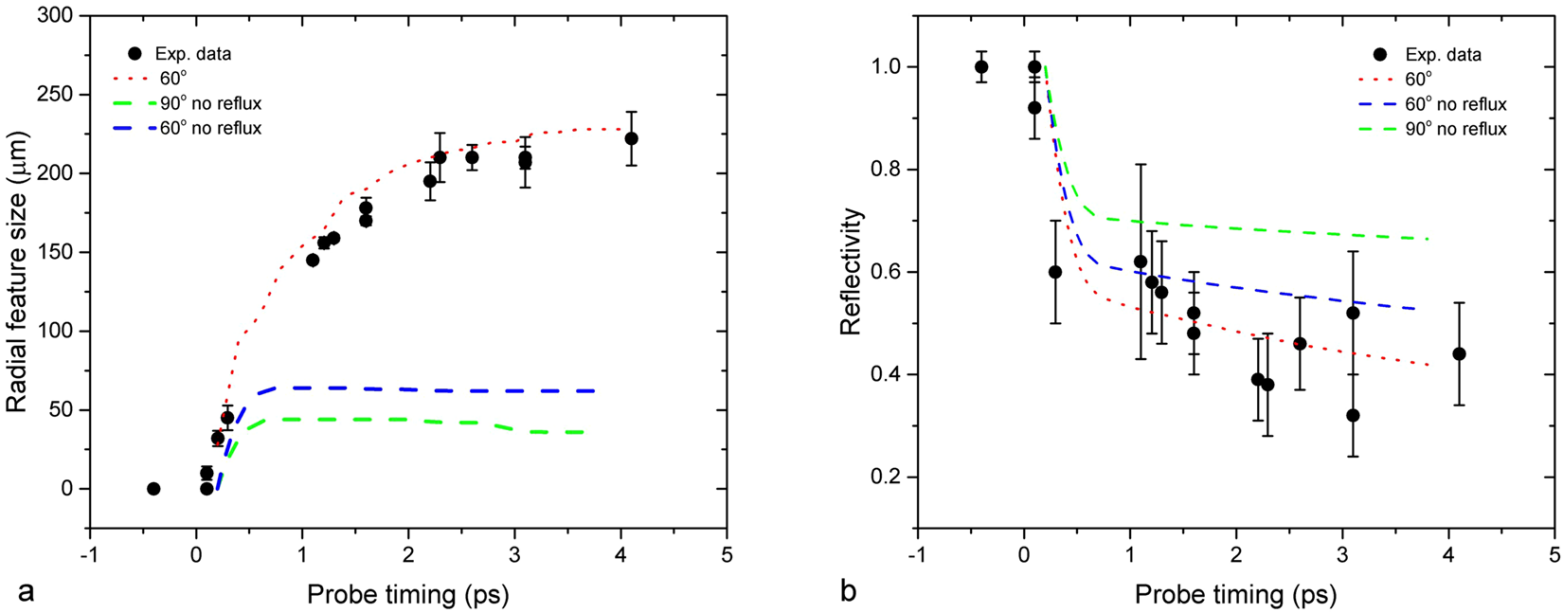
Summary of Zephyros simulation results with and without electron recirculation: (**a**) Radial measurement of area of reduced reflectivity as a function of probe time (**b**) Minimum reflectivity recorded as a function of probe time.

Figure 4 shows a plot of rear surface bulk temperature and the reflectivity calculated from the Zephyros simulations for multiple time steps. For the case where electron recirculation is not inhibited the expanding heated region (with a peak temperature of 0.8 eV) and corresponding area of dark reflectivity seen in the experiment are in good agreement, with a minimum reflectivity decreasing with time. Figure 5a shows the characteristic radial size of the simulated probe reflectivity at the rear surface, along with the corresponding experimental results. The characteristic rapid expansion at early time is followed by a slower expansion at later times, as observed experimentally.

In order to clarify the role of electron recirculation in driving the radial expansion observed with the diagnostic, a repeat simulation was performed with recirculation disabled. This was achieved by creating open boundaries on all sides of the simulation box, representing a model whereby only the first pass of any injected fast electrons would contribute to the bulk heating of the Aluminium foils. This simulation result is also shown in Fig. 5. While a noticeable drop in reflectivity is still observed, the radial expansion is severely limited to a maximum radius less than 30% of that measured experimentally. When the divergence half angle is increased to 90 degrees (Fig. 5) to maximise the radial spread of the electron population on a single pass, the degree of radial expansion and heating is further decreased. This can be accounted for by an insufficient electron number density at the target rear surface being no longer sufficient to produce clearly observable drops in reflectivity. Hence the sustained lateral expansion of the fast electron population and target heating measured experimentally can be seen to result from a combined effect of an initially broad electron distribution and the fact that a large part of the electron population is trapped within the target. After ≈2*ps* the simulations show that little additional target heating occurs in the central region of the target. At this point any further drop in reflectivity is accounted for by expansion of the heated plasma rather than a change in resistivity.

## Discussion

By utilising a high spatial and temporal resolution optical probe we have made measurements of fast electron driven lateral energy transport in the ultra-intense, short-pulse regime for the first time. Measurements of the target rear surface within a few picoseconds of the 10^21^ Wcm^−2^ drive laser reveal rapid radial expansion (≈0.5c) of a heated region which evolves long after the initial pulse length. Hybrid-PIC simulations of the interaction indicate that a large initial fast electron divergence angle coupled with electron recirculation are required to maintain the radial expansion and heating observed.

These measurements clearly impact many areas relating to laser-plasma acceleration and secondary sources. A large divergence angle coupled to electron recirculation could be beneficial, for example in enhancing X-ray flux (in a similar way as reported by Quinn *et al*.^24^). However applications may be limited if the X-ray source size were to grow too large and limit spatial resolution. From these experimental results and associated simulations, it is clear that for certain interaction conditions key parameters such as fast electron number density or bulk temperature, cannot be assumed to be derived directly from a single pass of the fast electron beam. Huang *et al*.^59^ and Sentoku *et al*.^18^ created analytical models for the threshold at which hot electron recirculation would have a significant effect on ion acceleration from the target rear surface. These models predict a threshold target thickness of ≈6–8 *μ*m for a pulse length of 40 fs. Since the optical probing technique used here is sensitive to small changes in fast electron driven target heating we have been able to measure the effect of electron recirculation for relatively thick targets. By applying the time-resolved diagnostic technique described in this article to a thinner target regime, it would be possible to investigate this transitional regime when coupled to complementary measurements of the ion beam.

Further experiments are needed to study key fast electron parameters and their subsequent effects on femtosecond laser-driven science and applications. Crucially we have demonstrated that time-resolved diagnostics are required to resolve aspects of electron generation and transport that may not be apparent with time-integrated observations. This work also highlights the requirement to extend developments relating to fast electron divergence control to the femtosecond regime, in order that the angular distribution can be brought within the tolerance range required for most secondary sources.

## Methods

The experiment was performed using the Gemini laser at the Rutherford Appleton Laboratory. Gemini is a dual-beam Ti:Sapphire laser system, consisting of two independently configurable beams lines, delivering 12 J with a pulse duration of ≈40 fs. The main (pump) beam was focused onto target at an incidence angle of 30 degrees using an f/2 parabola, with a resulting focal spot diameter of 3 *μ*m in a Full Width Half Maximum (FWHM). Accounting for losses in the system, the energy within this FWHM was up to 3 J, with a calculated peak intensity of 10^21^ Wcm^−2^. The laser contrast (the ratio of the peak laser intensity to the amplified spontaneous emission (ASE) pedestal intensity) was measured to be ≈10^8^ up to 20 ps before the main pulse.

A 10 mm apodised portion of the second Gemini beam was used as a rear surface optical probe, incident at an angle of 40 degrees relative to target normal in a p-polarised orientation. The beam was chirped to a pulse length of between 0.3 and 5 ps by detuning the compressor. At the shortest pulse duration (300 fs) the temporal resolution was 100 fs. By using a linear chirp, a linear relationship between wavelength and probe timing could be assumed, the shortest wavelengths (≈780 nm) probing earliest in time, the longest (≈820 nm) latest in time. In order to separate the time windows, three CCD cameras were using in the imaging line, with a different bandpass filter in front of each camera (see Green *et al*.^41^ for more details). An f = 40 cm achromatic lens was used to image the target rear surface, yielding a spatial resolution of 6 *μ*m at 800 nm, and a magnification of ×7.

The timing of the rear surface probe relative to the main pulse was also varied with the use of a timing slide. The intensity of the rear surface probe was ≈5 × 10^10^ Wcm^−2^, an intensity that was found to have no measurable effect on the target rear surface itself, even for ultrathin (<100 nm) targets. In order to make quantitative measurements of the rear surface reflectivity a reference image of each target was first obtained by just firing the probe beam of the Gemini laser. A second image was then taken with both the probe beam and main interaction pulse incident on the target, with the timing between the two varied on each shot. By normalising the on-shot reflectivity map with the reference reflectivity map obtained from the undisturbed foil, a clear 2D measurement of any change in target reflectivity was obtained.

The datasets generated during and/or analysed during the current study are available in the STFC Research Data Repository, http://dx.doi.org/10.5286/edata/712.
